# From St. John’s wort to tomato and from Rhodiola to cranberry: A review of phytotherapy and some examples

**DOI:** 10.1007/s00508-020-01633-w

**Published:** 2020-03-24

**Authors:** Verena Weixlbaumer, Lukas Draxler, Markus Zeitlinger, Benedikt Prantl

**Affiliations:** 1 Department of Clinical Pharmacology, Vienna University Hospital, Medical University of Vienna, Waehringer Guertel 18–20, 1090 Vienna, Austria; 2 Medical University of Vienna, Vienna, Austria

**Keywords:** Phytotherapy, Randomized controlled trial, Evidence, Traditional medicine, Food supplement

## Abstract

**Background:**

This study evaluated the definition, the prevalence of use and the governmental regulations of phytotherapy and four examples of herbal medicine are discussed in more detail.

**Results:**

This research group evaluated 4 topics: St. John’s wort for treating (mild to moderate) depression, tomato extract as a platelet inhibitor, Rhodiola against stress-related fatigue and cranberries for the treatment of urinary tract infections.

**Conclusion:**

The findings were diverse and must be individually taken into account. Evidence for efficacy varies within and between the four examples. An explanation for the lack of reproducibility of findings from preclinical and clinical experiments might be the insufficient standardization of herbal medicines. There is no scientific reason why phytotherapy should not be investigated with the same rigor as conventional drugs to establish the efficacy and potential risks. Meanwhile, it is concluded that care is essential when using herbal medicine in the daily routine and informing patients about potential shortcomings and dangers of herbal medicines should be considered a duty of pharmacists and physicians.

## Introduction

As part of a university course, student groups were created to analyze different segments of complementary or alternative medicine in order to evaluate the evidence for efficacy and safety in the respective field. This short review presents the result of the group which focused on phytotherapy, an area where evidence-based and complementary medicine meet each other.

## Definition and history

Phytotherapy or herbal medicine defines a broad spectrum of treatment combining modern and traditional knowledge of plant-based mixtures of several potentially active substances. Regulations by national (e.g. Austrian) and European authorities, such as the EMA split these substances into two classes: medicinal products and dietary supplements.

Modern phytopharmaceuticals contain defined amounts of biologically active compounds ensuring consistency in the amount of active substances. Additionally, if the product has been approved as a drug, safety and efficacy need to have been demonstrated in preclinical and clinical tests before approval. These standards are described in monographs containing detailed information about pharmacovigilance, clinical and non-clinical data, giving national competent authorities “[…] one unique set of information on a herbal substance or preparation when evaluating marketing applications” [1]. Further information regarding historical data, national regulatory status overview and herbal preparation are also available [2].

Monographs in the European Union (EU) made by the Herbal Medicinal Product Committee (HMPC) serve as orientation for pharmaceutical companies and national registration/admission authorities and correspond to the information presented in the summary of product characteristics (SmPC).

The estimated market for herbal medicine in Austria (2018) alone amounts to 300 million € or 7% of the total revenue of the pharmaceutical industry [3].

The medical use of herbs should not be considered as a human achievement only. Studies showed that even chimpanzees (*Pan troglodytes*) practise self-medication by using indigestible remedies, such as *Vernonia amygdalina *against intestinal parasites [4].

Natural herbs historically represented a more rational alternative to conventional medicine, which for centuries included methods, such as bloodletting, cupping and dirt-pharmacy with virtually no benefits and fatal side effects. In the nineteenth century, during the dawn of modern pharmacy, plants, such as the opium poppy, willow bark or coca leaf contributed essential basic material for important medicines (e.g. salicylate, morphia, cocaine).

In 1913, Henri Leclerc published “Précis de phytothérapie” in which he sought to differentiate between traditional herbal therapies and modern plant-based medicine for the first time by creating the term phytotherapy.

Herbal medicine declined with the growth of economic and technological possibilities, but it was resurrected in times of resource scarcity or in socioeconomically deprived areas, and continues to be practiced in many societies worldwide [5, 6].

Nowadays, the World Health Organization (WHO) traditional medicine strategy 2014–2023 acknowledges the importance of traditional medicine systems in local primary healthcare, relying commonly on herbal medicines, rather than replacing it with fragmentary or insufficient western standards [7]. “The strategy aims to support Member States in developing proactive policies and implementing action plans that will strengthen the role traditional medicine plays in keeping populations healthy” [8].

While many traditional plant-based remedies have shown no clinical effects in laboratory studies, there have been some notable successes. For example, the 2015 Nobel Prize for Medicine was awarded to Chinese pharmaceutical chemist Tu Youyou for her contribution to the discovery of artemisinin in curing malaria. She discovered the substance through a survey of traditional Chinese medicines, which shows that the cooperation between modern conventional medicine and scientific phytotherapy can be worthwhile [9].

## Herbal preparations

The administration of herbs consists of a variety of pharmaceutical preparations, the most common of which are liquids (e.g. infusions or herbal teas) and decoctions. Given that certain components dissolve at a particular time, the preparation is time-dependent, and some require the usage of cover plates due to the etheric oils which are highly volatile. The formulations follow the characteristics and galenic needs of the ingredients and active components which are to be extracted from the plant parts [10].

Liquid preparations can be further subclassified into fresh plant preparations, tea preparations and extracts. Each of these are further subdivided according to their method of production, as described below.

Fresh plant preparations can be freshly squeezed juices, distillates and oil-based extractions. Water-vapor volatile ingredients are gathered through steam distillation; however, for oil-based extractions, plant parts are inserted in oil at moderate temperature to extract the liposoluble components.

Next, tea preparations can be further subdivided into infusions, decoctions and macerations. For infusions, chopped plant parts are doused with hot water, steeped and strained after a specific brewing time. This is mostly used for volatile oils and thermolabile ingredients. More solid parts like bark or lumber are extracted through decoctions. These are made by boiling and simmering the plants in water. Maceration is mostly used with plants that would release unwanted ingredients or gelatinize when combined with hot water (e.g. marshmallow root). In such cases, chopped plant parts are steeped in water at room temperature for several hours.

Finally, extracts are further divided into alcoholic extracts, tonic preparations and alcoholic distillates. They usually exist in a liquid form, but it is also common to gradually evaporate the extraction medium. In doing so, a semifluid extract is produced. If the extraction medium is reduced to 4% residual moisture, a dry extract remains.

Semisolid preparations are further processed mostly into baths, jellies, pastilles, salves, syrups and suppositories [10]. Dry extracts, shredded and pulverized drugs can be further processed into dragées, granulates, gelatine capsules, pastilles and pellets. The quality of this dry preparation depends on the extraction medium, the extraction procedure and the drying technique [10]. The desired concentration can be gained by adding inert substances (e.g. lactose, dextrin) or mixing different extract charges [14, 15].

Alcoholic extracts known by patients as “drops” are usually “tinctures” or “fluid extracts”. Ethanol is used as an extraction medium with an alcoholic content of 20–60% by volume. Therefore, they are contraindicated in alcoholic patients and those with severe liver diseases. The ratio between drug and extraction medium in tinctures is typically between 1:5 and 1:10 and sometimes has to be diluted before use. Fluid extracts have a 1:1 ratio and hence are of higher concentration.

Ultimately, the last form of liquid preparations is in the form of alcoholic distillates. The alcoholic content has to be at least 40 vol.% to provide a clear dissolution of the extracted aetheric oils [10–12].

In relation to extracts the drug-extract ratio (DER) must be mentioned, a fundamental parameter for the comparability of extracts. It is a measurement for the extract yield in a standardized procedure depending on the used plant parts, the extraction medium and the extraction method [13, 14].

Normally, only plant-based drug products with a relatively constant composition should be used in different preparations. Drugs made from self-collected or self-cultivated plants may or may not have the desired activity but carry additional risks of allergic reactions due to contamination or overdosing with associated side effects.

The most common forms of application are perioral intake or ointments because intravenous phytotherapies carry potential risks, such as clouding and sedimentation [11, 14, 15]. Moreover, severe infections have been described after application of parenteral phytotherapies.

### Governmental regulations

#### Authorization of phytopharmaceuticals in the European Union

There are several procedures for authorizing drugs in the European Union [16].

1. In a national approval, drugs are only authorized in one of the member states. In Austria, this is incumbent upon the Austrian Ministry for Safety and Healthcare System (*Bundesamt für Sicherheit und Gesundheitswesen*, BASG).
2. If there is an authorization in one of the member states, the drug can be authorized through the procedure of mutual recognition, whereby other states can authorize the drug in a simplified procedure.
3. If there is no authorization in any member state, usually a drug has to be authorized in all members states at the same time by a centralized approval via the EMA. For modern drugs this is by far the most important pathway and decentralized procedures are only possible under certain exemptions.

### Licensing a phytopharmaceutical in the European Union

There are 3 ways of licensing a phytopharmaceutical: traditional use registration, well-established use marketing authorization and the stand-alone or mixed application [17].

1. The traditional use registration applies for phytopharmaceuticals which have been used for at least 30 years, 15 of which in the European Union. Approval involves an assessment of mostly bibliographic safety and efficacy date. Clinical tests are not necessary as long as sufficient data concerning safety and plausible effect are available from the literature. Herbal medicine brought to the EU market via the traditional use registration are not intended to be used with the supervision of a medical professional and must not be administered by injection. In the Austrian Medicinal Products Act (*Arzneimittelgesetz*, AMG) the traditional use registration corresponds to § 12.
2. The well-established use marketing authorization applies if literature indicates that the active substances has been in well-established use within the EU for at least 10 years, with recognized efficacy and an acceptable level of safety. This involves an assessment of mostly bibliographic safety and efficacy data. In the AMG the well-established use registration corresponds to §10a.
3. The stand-alone or mixed application needs the same registration procedure as any other commercial drug. The manufacturer has to prove the efficacy and safety in preclinical and clinical studies.

A clear distinction has to be made between phytotherapies marketed as pharmaceuticals and those sold as dietary supplements with a botanical background. In Austria, phytopharmaceuticals are regulated within the AMG and are therefore drugs by definition. On the other hand, dietary supplements are regulated by the Food Safety and Consumer Protection Act (*Lebensmittelsicherheits- und Verbraucherschutzgesetz*, LMSVG); where they are controlled similarly to standard food products.

The requirements for dietary supplements are much lower, which seems to be one key factor for the difference in the quality of these products.

### Licensing on the nation level in Austria: the Medicinal Products Act (*Arzneimittelgesetz*, AMG)

In the Austrian Medicinal Products Act § 1. (1) a drug is defined as “a substance, used in or on the human or animal body, with the purpose of healing, relieving or preventing suffering from human and animal disease or pathological discomfort or which is supposed to be used in or on the human or animal body or which is supposed to be administered to a human or an animal to either restore, correct or influence physiological functions through pharmacological, immunological or metabolic effect or to be used as the basis for a medical diagnosis.”

In § 1 (3) the AMG defines what does not qualify as a drug. In § 1. (3) 2. dietary supplements are explicitly mentioned, as not being part of the definition of a drug. In § 1. (3) 9. all substances that are planned to be used exclusively as complementary medicine, if they are not fulfilling the definition in § 1 sect. 1, are excluded from the definition of a drug. Not included in this exclusion are all substances which are produced under homeopathic principles.

## Examples of phytotherapy and the evidence

After a literature review the following examples were bibliographically investigated:

### Cranberry

Cranberries are low evergreen subshrubs which can be found in chilly areas of the northern hemisphere in acidic bogs [18]. It is a popular opinion that cranberry products can help when suffering from urinary tract infections (UTI). They are one of the most common reasons for antibiotic treatment and due to the spreading of resistance against first line antibiotic therapies, there is public demand for non-antibiotic treatment alternatives for these infections [19, 20].

The main focus regarding non-antibiotic UTI therapy rests on the usage of various food and medical grade cranberry products, but there are other therapies discussed as well (e.g. vitamins, immunotherapy, probiotics, NSAIDs [non-steroidal anti-inflammatory drugs], D‑mannose and estrogens) [20].

In the scientific literature, some studies reported a thoroughly positive effect, whereas others showed that cranberries have no significant impact on UTI treatment or prevention [20, 21]. Multiple reasons might exist why it is still unknown whether UTI therapy using cranberries is effective. One of the main problems is the inconsistency in methods used in clinical studies; variability in choice of primary outcome, standardization of intervention, study design and inclusion/exclusion criteria limit the value of the available meta-analyses on this subject [21].

Other common problems are the high risks of bias, such as selective outcome reporting and high loss to follow-up, as well as a lack of standardization in the different cranberry products concerning dosage and composition [22, 23].

Ex-vivo research showed that A‑type proanthocyanidins and other polyphenols have an anti-adhesive impact on bacteria but achieving sufficient concentration of these ingredients in the urinary tract seems unlikely [23].

Speculated synergistic effects between the different cranberry phenolic metabolites needs further analysis in light of patient variations of their metabolism [23].

To summarize, scientifically valuable studies or meta-analyses with focus on the most relevant populations have yet to be conducted. To confirm the findings, larger high-quality studies are required [21–23].

### *Rhodiolae rosea*

For arctic root (*Rhodiolae rosea*) different clinical [24, 25] and non-clinical [26, 27] effects have been described over the last centuries. *Rhodiolae* is referred to as a well-distributed adaptogen [28] and is generally used for reduction of stress-related syndromes, such as fatigue and burnout [29]. The perennial plant in the family *Crassulaceae* is part of traditional medical culture in Russia (zolotóy kóren), Scandinavia (rosenrot) and Germany (Rosenwurz). An expanding market for Rhodiolae supplements, pushing the registration of EMA approved, non-prescriptive rhodiolae extracts in Switzerland (Vitango® 2010, Schwabe Pharma AG, Küssnacht, Switzerland) or Sweden (SHR-5® 1985, Artic Root®, Swedish Herbal Institute, Gothenburg, Sweden), meet patients’ demand for natural remedies [30].

Nootropics (food supplements), which promise feeling “high”, curing cancer and heart ailments, are the downside of this “natural trend”, since there are no reliable data confirming these effects [31].

The EMAʼs Committee on Herbal Medicinal Products concluded that: “On the basis of its long-standing use, arctic root can be used for the temporary relief of symptoms of stress, such as fatigue and sensation of weakness.” but “[the] results from trials on clinical pharmacology are contradictory. The number of clinical trials for clinical efficacy is limited, also the number of included subjects” [32].

In the following section, one specific randomized clinical trial, as a role model for available studies is discussed. The study [33] investigated the effects of SHR‑5 root extract on attention, quality of life, saliva cortisol response to awakening, symptoms of fatigue and depression in subjects with stress-related fatigue. In this parallel group study, 60 patients were randomly assigned to 2 daily doses of either 390 mg verum (including 144 mg 5SHR extract) or a 390 mg placebo. The effects of the extract were verified on the basis of classical scoring (e.g. Montgomery depression score, Pine’s burnout scale, SF36 and Conner’s computerised continuous performance test II, saliva cortisol response). These were assessed on day 1 as well as on day 28. The results showed statistically significant improvements concerning Pine’s burnout scale, cortisone reduction, and attention compared to the placebo group. The authors concluded that the extract SHR‑5 exerts an anti-fatigue effect that increases mental performance, particularly the ability to concentrate; however, the positive change in depression scores over time for both groups (placebo + control) in mild depression (MADRS [Montgomery–Åsberg Depression Rating Scale]), mental and physical health (SF36) seem to be related to regression toward the mean.

### St. John’s wort

One of the most famous representatives of phytotherapy is St. John’s wort (*Hypericum perforatum*). The herb, which has small clusters of yellow flowers that release a dark red oil when crushed, is used by many cultures around the world to treat depressive mood and wound healing disorders. In recent decades, due to the increased prevalence of depression, researchers have sought to clarify the pharmacological mechanism of action of the individual compounds of the herb (*Hypericum* extracts), to further enable development of drugs for depression.

Research teams have independently addressed the questions of how St. John’s wort plays a role in the treatment of depression, and whether it can be used as an alternative to known SSRIs (selective serotonin reuptake inhibitors). The results of the studies were contradictive, and systemic reviews were performed in order to summarize their findings. In 2005 Linde et al. published a meta-analysis in the British Journal of Psychiatry, in which they included 37 double-blind randomized controlled trials that compared clinical effects of *Hypericum* with either placebo (26 trials) or standard antidepressant drugs (14 trials) in adults with depressive disorders. The meta-analysis found that in smaller trials, *Hypericum* extracts showed marked effects over placebo, whereas in larger trials, the effects were minimal. Compared with standard antidepressants the authors concluded that the evidence regarding *Hypericum* extract is inconsistent and conflicting. In double blinded placebo controlled trials for patients with major depression disorder *Hypericum* extract showed only minimal beneficial results, whereas active controlled trials found similar effects to standard antidepressants (which means that *Hypericum* extracts were either equally effective or ineffective) [34].

Approximately 4 years later Linde et al. again reviewed 29 studies with 5489 patients with depression that compared treatment with extracts of St. John’s wort for 4–12 weeks with placebo treatment or standard antidepressants in another systemic review. The authors concluded that *Hypericum* extract is superior to placebo and not significantly inferior to standard antidepressants. Moreover, they concluded that there were fewer side effects in the *Hypericum* group, compared to the standard antidepressants. An interesting finding was that all trials from German-speaking countries reported findings more favorable to *Hypericum* than from other countries of origin [35].

Nga et al. analyzed in their meta-analysis 27 clinical trials with a total of 3808 patients, comparing the use of St. John’s wort and SSRIs for patients with depression. The authors concluded that for patients with mild to moderate depression, St. John’s wort has comparable efficacy and safety compared to SSRIs. Again, the discontinuation of treatment due to side effects was lower in the *Hypericum* group. They also noted that follow-up studies carried out over a longer duration should be planned to ascertain its benefits [36].

Nga et al. and Linde et al. both described non-inferiority to standard antidepressant when treating mild to moderate depression. They also found that *Hypericum* has fewer side effects which led to lower subject attrition. A meta-analysis of 19 studies reported that the discontinuation rate for standard SSRIs is around 25.6%, with 10.8% attributed to side effects [36, 37].

It should be mentioned that even if St. John’s wort is not inferior to SSRIs when treating depression, this does not necessarily make it effective, since the efficacy of SSRIs and other antidepressants might also be questioned in some forms of depression. Furthermore, the procedure of preparing (preparation) and the origin of the St. John’s wort seems poorly standardized, which hampers comparison of different studies with one another.

### Tomato extract and its effect on platelet function

Frequently, phytotherapeutics are products with a herbal or botanical background which are licensed, marketed and sold by pharmaceutical companies as alternatives to established drugs. In this context, tomato extract was proclaimed as an alternative to aspirin and sold under the brand name Fruitflow®.

The group analyzed one publication, in which the authors report that 97% of the tested subjects experienced a platelet-inhibiting effect when taking tomato extract and compare this to approximately 25% of patients who have been reported to be resistant to the effects of aspirin in a similar setting [38].

In this study, the term “aspirin resistance” was used to describe a number of different phenomena including the inability of aspirin to: (i) protect individuals from thrombotic complications; (ii) cause a prolongation of the bleeding time; (iii) inhibit thromboxane (TX) biosynthesis; or (iv) produce an anticipated effect on one or more in vitro tests of platelets [39]. To assess the effects of tomato extracts as a dietary supplement to prevent platelet activation, a double blinded placebo-controlled crossover study was performed. In the study, 93 healthy human subjects, aged 45–70 years, with 40 women and 50 men were included (3 were withdrawn during the study). They were either given a high dose of tomato extract (6 TE = equivalent to the amount of antiplatelet components quantified in 6 fresh tomatoes), a low dose tomato extract (2 TE) or a placebo.

The responder rate, which led to the total of 97% responders, was defined as a difference in response of the platelets to ADP (adenosindiphosphate) at different concentrations or to collagen. The results for the responder rates are in Table 1.

**Table 1:** Responder rate after in-vitro challenge with adenosindiphosphate (ADP) or collagen after intact of two different doses of tomato extract (TE)

	6 TE (%)	2 TE (%)
3 µmol ADP/L	61	51
7.5 µmol ADP/L	50	45
3 mg Collagen/L	52	45

*TE* tomato extract

All individual responder rates are close to 50%, which could be explained by the fact that the odds of a test result increasing or decreasing when being repeated are 50% under neutral conditions, i.e. like tossing a coin. Only 3% of patients did not react to any of the 6 tests, falsely creating a responder rate of 97%.

Fruitflow® is marketed as an alternative to aspirin, but is only a dietary supplement, not a drug. Therefore, it was approved only by the European Food Safety Authority, and not by the EMA.

We think that it is dangerous to present advertisements in a seemingly scientific way using improperly reported study data. Physiologically active, evidence-based, and necessary treatment might be withheld from subjects who seek a “natural” alterative to chemically produced “industry” drugs due to misleading marketing strategies.

## Conclusion

This review considered just four examples of herbal medicine with very different levels of dissemination but also different levels of evidence. We picked these diverse examples explicitly to show the wide range of efficacies and popularities of various herbal remedies and encourage both an open mind and critical eye when it comes to this field.

Herbal medicine sometimes might bring hope to patients, when there seems to be no treatment available within the evidence-based medicine. Human mind patterns often tend to deal in absolutes: with many either choosing to hastily embrace traditional herbal medicine ancient wisdom, or reject the entire field as pseudoscience. Realistically, much of modern medicine has been built from plant isolates, and many remedies likely still exist in nature; still, care must be taken to identify these treatments utilizing good scientific practices and ensuring the highest standards of patient health and safety.
